# Supplementary material for: A human-machine interface for automatic exploration of chemical reaction networks
Source: Nat Commun. 2024 May 1;15:3680. doi: 10.1038/s41467-024-47997-9 (PMC11063077; doi:10.1038/s41467-024-47997-9)
Supplement: Supplementary file 1 — Supplementary Information [file 41467_2024_47997_MOESM1_ESM.pdf]

SUPPORTING INFORMATION

A human-machine interface for automatic exploration of chemical reaction networks

Miguel Steiner[a, b] and Markus Reiher[a, b, ‡]

[a] ETH Zurich, Department of Chemistry and Applied Biosciences, Vladimir-Prelog-Weg 2, 8093 Zurich, Switzerland
[b] ETH Zurich, NCCR Catalysis, Vladimir-Prelog-Weg 2, 8093 Zurich, Switzerland

[‡]Corresponding author; e-mail: mreiher@ethz.ch

1 Technical summary of the explorations

All explorations have been carried out with a development version of Chemoton 3.1 [*1*] in Heron [*2*]. The catalytic systems, the explored networks, and the required resources in terms of computing time are summarized in Supplementary Table 1.

In that table, the notation is as follows (in accordance with how we used it in this work): 'Serial computing time' denotes the collective serial computing time and given by the sum of the run time of all reaction explorations (excluding the refinement calculations with DFT) times the number of computing cores per calculation (on an AMD EPYC 7742 central processing unit). A structure is a three-dimensional arrangement of nuclei with a given total molecular charge and spin multiplicity. A compound is a set of molecular structures with the same nuclear composition and connectivity. Elementary steps connect one or more structures to one or more different structures via transition state structures. Reactions connect one or more compounds to one or more other compounds. For more details on these definitions, we refer to Ref. *3*.

The total serial computing time (*i.e.,* the time for running all calculations on a single processor core only) was 197 days for the reaction explorations based on semiempirical calculations for the three homogeneous explorations combined and 1272 days for the gallium single-site catalyst. The gallium single-site catalyst required considerable more computing time due to the complexity of the system, the depth of the reaction network (more than 30 exploration steps). The three other reaction networks included broader steps and exploited the embarrassingly parallel nature of our reaction network explorations [*4*]. These systems could be explored in about a single day with a moderate number of 100 processing cores on AMD EPYC 7742 central processing units.

The refinement of reaction and activation energies based on DFT required 632 days and 332 days of serial computing time for the exploration of the Monsanto process and the gallium single site catalyst. The refinement of the Monsanto process required more computing time, because individual calculations were carried out on multiple cores, decreasing the nominal efficiency. In general, the refinement is, however, embarrassingly parallel, because all refinement calculations can be carried out in parallel. One can estimate the computational time saved by our steered exploration approach by comparing the calculations with the steering protocol to the number of calculations required in an exhaustive approach that would cover the same chemical space. For this, we have taken all `Selection Steps` of the steering protocol for the Monsanto process exploration and combined them within a logical *'or'* superset, *i.e.,* a compound and its nuclei were considered reactive if *any* of the applied

`Selection Steps` selected them.

We then launched an elementary step gear equipped with an aggregate and reactive site filter generated from that super-set and imposed limits on the gear (in terms of the number of allowed bond modifications, bond formation reactions, and dissociation reactions) equivalent to the largest limit in all of the `Network Expansion Steps` in our steering protocol. Running this setup indefinitely would eventually cover the same chemical reaction space as our steered exploration. However, this approach would require 50,000,000 reaction trials compared to the 47,000 reaction trials that were necessary to explore the Monsanto process in a steered approach. Hence, we can estimate a 1,000-fold acceleration of our automated explorations. Therefore, our algorithm allows one to study complex reaction mechanisms in a general and exhaustive manner within days, compared to years in a brute-force manner, with little to no domain knowledge of the setup of quantum chemical calculations.

Supplementary Table 1: Overview of resources required and reactions found for three catalysts (first column). Note that the times significantly reduce in practice through parallelization, which is not visible in these serialized timings. Source data are provided as a Source Data file.

Catalyst	Reaction	#Compounds (#Structures)	#Reactions (#Elementary Steps)	Serial computing time / days
Wilkinson	Hydrogenation	80 (1051)	90 (370)	50.6
Ziegler–Natta	Polymerization	472 (3128)	291 (731)	5.6
Monsanto	Carbonylation	5465 (36796)	4987 (11751)	141.2
Gallium silicate	Polymerization	1795 (37053)	4533 (14118)	1272.2

2 Summary of the implemented exploration steps

Supplementary Table 2: Currently implemented `Network Expansion Steps`. Source data are provided as a Source Data file.

`Network Expansion Step`	Description
`Simple_Optimization`	Sets up structure optimizations; only meant to be applied after inserting new structures into the database

Network Expansion Step	Description
Thermochemistry_Generation	Carry out Hessian calculations on stationary point structures in the network
Conformer_Creation	Carry out conformer generation with MOLASSEMBLER [5]
Association	Carry out association reactions, *i.e.,* limiting reaction trials to bimolecular reactions
Dissociation	Carry out dissociation reactions, *i.e.,* limiting reaction trials to unimolecular reactions with only dissociative reaction coordinates
Rearrangement	Carry out rearrangement reactions, *i.e.,* limiting reaction trials to unimolecular reactions with a mix of associative and dissociative reaction coordinates

Supplementary Table 3: Short description of the implemented `Selection Steps`. The term 'aggregate' refers to a compound or flask within a CRN. Both are defined as a set of structures with the same nuclear composition and connectivity. Compounds and flasks differ in that flasks contain structures which have a disconnected graph. Source data are provided as a Source Data file.

Selection Step	Description
All_Compounds	No compounds are excluded.
Predetermined	Selection that returns a result without evaluating the database. This selection exists for the case that two expansion steps should be applied to the same selection. In this way, an expansion step A can be applied to a selection x, then this `Predetermined` selection can follow in order to proceed with the result of x, such that the next expansion step B receives the same selection result as the expansion step A.
File_Input	Inserts structures from a local file into the CRN. This is designed for starting an exploration.
Scine_Geometry_Input	Inserts structures from within a Python script or from SCINE INTERACTIVE within HERON into the CRN. This is designed for starting an exploration.
All_From_Previous_Result	Select everything that the previous expansion step produced
All_User_Inputs	Select all aggregates that were originally inserted into the network

Selection Step	Description
`Barriers_Within_Range`	Select all aggregates and structures that are products of the reactions in the previous expansion and have a barrier below a given threshold specified when adding the selection to the protocol.
`Lowest_Barrier`	Select the n lowest barrier aggregates and structures from the previous expansion with n being a parameter specified when adding the selection to the protocol.
`Products`	Select all aggregates and structures that are products of the reactions in the expansion
`Central_Metal`	Select aggregates according to a specified element, for instance a transition metal atom; carry out reactions with one species including that element and restrict active sites to those in its vicinity
`Centroid_Conformer`	Select the centroid of each aggregate
`Lowest_Energy_Conformer`	Select the lowest energy structure of each aggregate
`Cluster_Centroid_Conformer`	Carry out a clustering of all structures of all aggregates and select the cluster centroids, *i.e.,* the structure with the smallest sum of root mean square deviations to all other structures within the cluster.
`Lowest_Energy_Conformer_Per_Cluster`	Select the lowest energy structure of each cluster of the structures of all aggregates

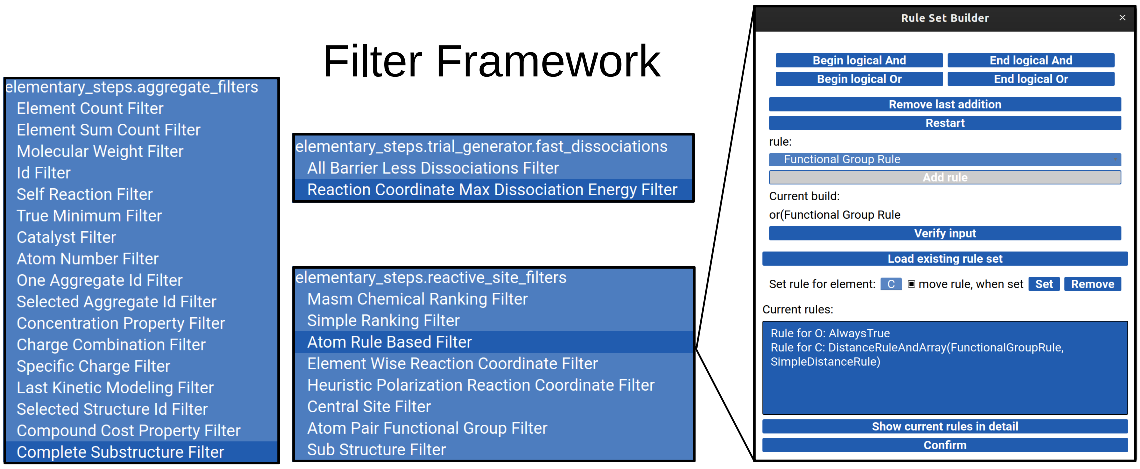

Supplementary Figure 1: Multiple screenshots of the menus in HERON that enable one to construct a custom set of instructions to filter out specific compounds or reactive sites. All implemented aggregate filters and reactive site filters in CHEMOTON can be selected, as explained in section 2.1 in the main text and Ref. *6*. On the right hand side, a set of graph distance based rules is constructed, which defines for each element a chemical surrounding defined by bond partners as either reactive or unreactive. Individual aggregate filters (left), reactive site filters (middle), and rules (right) can be combined with the logical operations '*and/or*', allowing a high degree of flexibility.

3 Screenshot of the Heron Interface

We present a simplified view of the graphical interface of the STEERING WHEEL in HERON in the main text in Fig. 2. This representation left out details that were not important for the overall structure and data flow in steering an exploration. Here, we include a screenshot of the graphical user interface to present the actual view in the program.

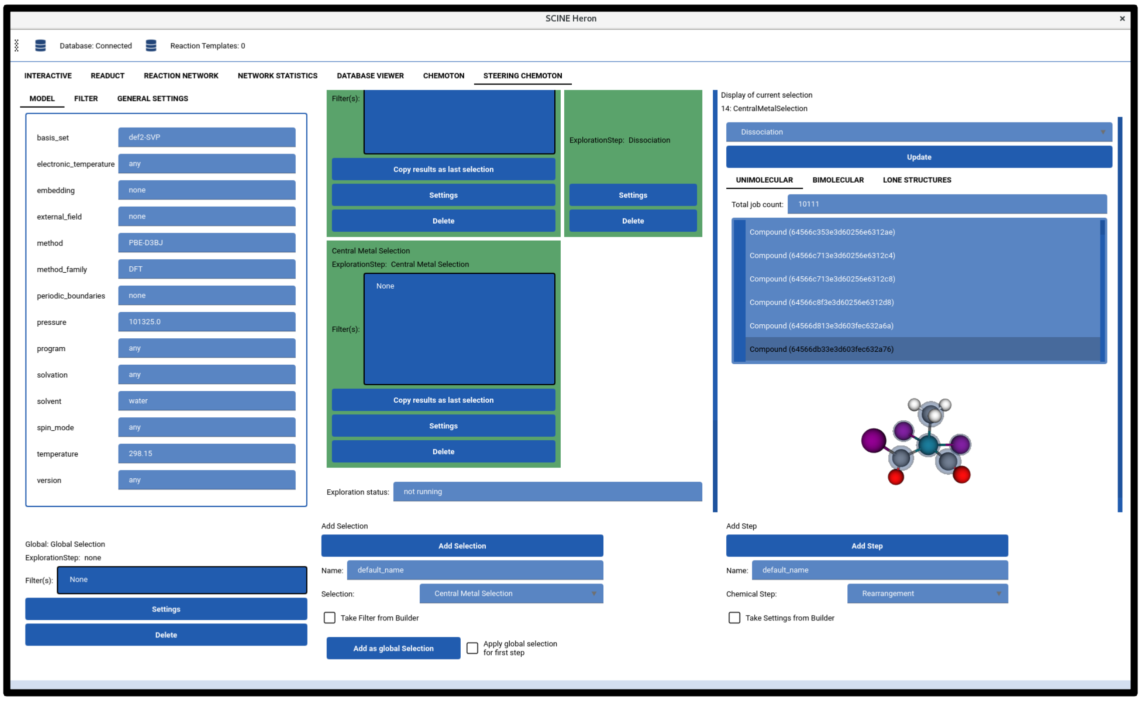

Supplementary Figure 2: A screenshot of the Steering Wheel interface in Heron. The tabs on the top provide similar windows for interactive manipulation of individual structure, analysis of the reaction network, and additional exploration control. The sub-tabs on the left allow one to select the electronic structure model, build specialized filters to specialize `Selection Steps`, and add user-defined settings to `Network Expansion Steps` (see tick boxes on the bottom). In the center, the exploration protocol with individual boxes for each exploration step is displayed. The green background color signals a successful execution. The right console allows one to query the latest `Selection Step` for a potential next `Network Expansion Step`. In this case, a `Dissociation`, an abbreviation for a dissociation reaction, was selected as a potential next expansion step, meaning that the selected subset of the reaction network is probed for dissociation reactions. All the resulting calculations that would be set up for this purpose are then displayed within that console. Each potential reactive complex can be selected to be visualized as a three-dimensional structure. The blue transparent spheres in this structure represent the reactive sites of the specific structure. The pull-down menus on the bottom then allow one to add the next exploration step to the steering protocol. A feature that is hidden in this view due to the scrolled-down view in the center, but is included in the schematic representation, is the possibility to safe and load exploration protocols.

References

[1] Bensberg, M.; Grimmel, S. A.; Lang, L.; Simm, G. N.; Sobez, J.-G.; Steiner, M.; Türtscher, P. L.; Unsleber, J. P.; Weymuth, T.; Reiher, M. qcscine/Chemoton: Release 3.0.0. 2023; https://zenodo.org/record/7928104.

[2] Bensberg, M.; Brandino, G. P.; Can, Y.; Del, M.; Grimmel, S. A.; Mesiti, M.; Müller, C. H.; Steiner, M.; Türtscher, P. L.; Unsleber, J. P.; Weberndorfer, M.; Weymuth, T.; Reiher, M. qcscine/Heron: Release 1.0.0. 2022; https://zenodo.org/record/7038388.

[3] Unsleber, J. P.; Reiher, M. The Exploration of Chemical Reaction Networks. *Annu. Rev. Phys. Chem.* **2020**, *71*, 121–142.

[4] Steiner, M.; Reiher, M. Autonomous Reaction Network Exploration in Homogeneous and Heterogeneous Catalysis. *Top. Catal.* **2022**, *65*, 6–39.

[5] Sobez, J.-G.; Reiher, M. Molassembler: Molecular Graph Construction, Modification, and Conformer Generation for Inorganic and Organic Molecules. *J. Chem. Inf. Model.* **2020**, *60*, 3884–3900.

[6] Unsleber, J. P.; Grimmel, S. A.; Reiher, M. Chemoton 2.0: Autonomous Exploration of Chemical Reaction Networks. *J. Chem. Theory Comput.* **2022**, *18*, 5393–5409.
